# Long-term endocrine complications and quality of life in pediatric-onset craniopharyngioma: a single-center cohort study

**DOI:** 10.1186/s12887-026-07309-0

**Published:** 2026-07-15

**Authors:** Phatwarin Kraiwiboonsit, Napat Sittanomai, Inthira Khampalikit, Thammanoon Surachatkumtonekul, Supitcha Patjamontri

**Affiliations:** 1 Department of Pediatrics, Faculty of Medicine Siriraj Hospital, Mahidol University, Bangkok, Thailand; 2 Division of Child and Adolescent Psychiatry, Department of Pediatrics, Faculty of Medicine Siriraj Hospital, Mahidol University, Bangkok, Thailand; 3 Division of Neurosurgery, Department of Surgery, Faculty of Medicine Siriraj Hospital, Mahidol University, Bangkok, Thailand; 4 Department of Ophthalmology, Faculty of Medicine Siriraj Hospital, Mahidol University, Bangkok, Thailand; 5 Division of Endocrinology and Metabolism, Department of Pediatrics, Faculty of Medicine Siriraj Hospital, Mahidol University, 2 Wang Lang Road, Siriraj, Bangkok Noi, Bangkok, 10700 Thailand

**Keywords:** Pediatric Craniopharyngioma, Quality of life, PedsQL

## Abstract

**Background:**

Craniopharyngioma is a benign yet locally aggressive brain tumor that causes substantial long-term endocrine, metabolic, visual, and hypothalamic morbidities, leading to impaired quality of life (QoL). However, data on QoL and long-term outcomes in Thai pediatric patients remain limited.

**Methods:**

This study included two components: a retrospective cohort review of long-term endocrine, metabolic, and visual outcomes in patients aged ≤ 25 years with histologically confirmed pediatric-onset craniopharyngioma diagnosed between 2000 and 2023 at Siriraj Hospital, and a cross-sectional assessment of quality of life using the Pediatric Quality of Life Inventory (PedsQL 4.0). Factors associated with quality of life were evaluated using linear regression.

**Results:**

Eighteen patients were included (median age at diagnosis 6.8 years). Postoperative endocrine deficiencies were common, including growth hormone deficiency (88.9%), vasopressin deficiency (100%) and gonadotropin deficiency (93.3% of evaluable patients). Residual/recurrent disease developed in 55.6% of patients during follow-up. Metabolic complications were common, overweight and obesity at recent follow-up were observed in 6 (33.3%) and 4 (22.2%) patients, respectively. Among screened patients (88.9%), dyslipidemia occurred in 62.5%, metabolic dysfunction–associated steatotic liver disease in 25.0%, and type 2 diabetes in one patient. Height SDS declined and BMI SDS increased significantly over time (*p* < 0.05). Visual impairment progressed in 27.8%. The mean PedsQL score was 68.8 ± 15.2. In exploratory analyses, obesity was associated with poorer quality of life.

**Conclusions:**

Pediatric craniopharyngioma carries a substantial burden of long-term endocrine, metabolic, and visual morbidity, adversely affecting growth and quality of life. Lifelong, multidisciplinary follow-up is essential to optimize outcomes.

**Supplementary Information:**

The online version contains supplementary material available at https://doi.org/10.1186/s12887-026-07309-0.

## Background

Craniopharyngioma is a rare epithelial tumor arising from remnants of Rathke’s pouch along the craniopharyngeal duct. It is typically located in the sellar and suprasellar regions, in close proximity to the pituitary gland and stalk, hypothalamus, optic apparatus, third ventricle, and major cerebral vessels. Tumor growth frequently results in mass effects that cause endocrine dysfunction and visual impairment, thereby increasing the complexity of surgical management [1]. The incidence of craniopharyngioma is approximately 0.5–2.5 new cases per 1,000,000 population per year globally, with 30–50% of these occurring in children and adolescents and accounting for 1.2–4.6% of pediatric intracranial tumors [2, 3]. Although overall survival is excellent, craniopharyngioma remains associated with high recurrence rates and considerable treatment-related morbidity, often resulting in long-term sequelae and impaired quality of life, particularly among long-term survivors [4]. The long-term burden of craniopharyngioma arises from both tumor progression and treatment-related effects. From one side, tumor recurrence may lead to serious, potentially life-threatening complications, while survivors frequently experience chronic endocrine deficiencies. Treatment-related hypothalamic injury can result in hypothalamic obesity and subsequent cardiometabolic complications, including type 2 diabetes mellitus, metabolic disorders, metabolic dysfunction–associated steatotic liver disease (MASLD), and hypertension. In addition, visual complications, including reduced visual acuity, visual field defects, optic neuropathy, and blindness, are common and may lead to long-term visual impairment [5–7]. The extent of neurosurgical resection in craniopharyngioma remains controversial. Over the past two decades, the traditional goal of gross total resection has increasingly shifted toward a hypothalamus-sparing approach that favors limited resection with or without adjuvant radiotherapy [8–10]. Contemporary treatment planning is largely guided by visual status and the extent of hypothalamic involvement on preoperative magnetic resonance imaging (MRI), with the aim of maximizing tumor control while minimizing long-term treatment-related morbidity [9, 11]. Although survival rates in childhood-onset craniopharyngioma are high, quality of life is often impaired by disease- and treatment-related factors, including neuroendocrine deficits such as growth hormone deficiency. The reduced quality of life in patients with craniopharyngioma is multifactorial [12]. Overall, childhood-onset craniopharyngioma is associated with reduced quality of life, particularly in emotional and social domains, which may adversely affect interpersonal relationships and social functioning [12–14]. Siriraj Hospital is one of the major referral centers for pediatric brain tumors in Thailand. This study aimed to evaluate endocrine complications, quality of life, and factors associated with quality of life among pediatric patients with craniopharyngioma treated at Siriraj Hospital.

## Patients and methods

This study consisted of a retrospective cohort study evaluating long-term clinical outcomes and a cross-sectional assessment of quality of life in patients with histologically confirmed pediatric-onset craniopharyngioma treated at Siriraj Hospital between January 2000 and December 2023. Patient data were obtained from electronic medical records. Pituitary hormone deficiencies were defined according to retrospective chart review. Growth hormone deficiency (GHD) was evaluated and diagnosed according to the following criteria: 1. Auxological data (short stature was height Standard Deviation Score (SDS) < −2.0 SDS) and/or height SDS < 1.0 SD below target height, growth velocity < 25th percentile for sex 2. Biochemical data (blunted response to growth hormone (GH) stimulation tests; clonidine and/or insulin, GH peak ≤ 7 ng/mL). Adrenocorticotropin (ACTH) deficiency was evaluated and diagnosed according to the following criteria: Low-dose ACTH stimulation test; peak cortisol < 15 µg/dL and/or morning cortisol < 3 µg/dL. TSH deficiency was evaluated and diagnosed based on low serum free thyroxine (FT4) levels with inappropriately low or normal TSH concentrations. Vasopressin deficiency was evaluated and diagnosed based on elevated serum osmolality/serum sodium with inappropriately low urine osmolality. Gonadotropin deficiency was established based on clinical signs of absent or incomplete pubertal development with biochemical confirmation with low sex steroids and inappropriately low serum gonadotropins. Height SDS and body mass index standard deviation score (BMI SDS) were calculated using the National Growth References for Children Aged 5–19 Years, 2020, Bureau of Nutrition, Department of Health, Ministry of Public Health, Thailand. Overweight was defined as BMI SDS > + 1 and ≤ + 2, while obesity was defined as BMI SDS > + 2. Blindness was defined as no light perception and diagnosed by an ophthalmologist. The term of gross total resection (GTR) was defined as complete excision of the tumor as documented by the operating neurosurgeon. In contrast, subtotal resection (STR) was defined as incomplete tumor removal, as documented in the electronic operative note. At our institution, surgical strategy evolved over time. GTR was pursued when complete excision was considered feasible without unacceptable risk, whereas tumors with significant hypothalamic involvement were more likely to undergo STR followed by radiotherapy in an effort to preserve hypothalamic function. Recurrence was defined as tumor regrowth detected on follow-up imaging after a postoperative MRI had confirmed the absence of residual disease. Residual disease was defined as persistent tumor identified on the first postoperative MRI. When early postoperative imaging was unavailable or delayed, lesions detected during follow-up were categorized as residual/recurrent disease because the distinction between residual and recurrent tumor could not be determined.

Quality of life was evaluated using the validated Thai-version pediatric quality of life (PedsQL 4.0) [15] for calculating the quality of life of these patients. PedsQL is a comprehensive, validated instrument consisting of four domains for assessing quality of life in children and adolescents aged 8–25 years. The 23-item PedsQL 4.0 Generic core scales include eight items assessing physical function and five items assessing emotional function, social function and school function. Parents of the patients were asked to complete the PedsQL 4.0 Generic core scales questionnaire.

### Statistical analysis

Data were analyzed using SPSS (Statistical Package for the Social Sciences for Windows, version 30.0). Categorical variables were summarized as frequencies and proportions, whereas continuous variables were summarized as means and standard deviations or medians with minimum and maximum values, as appropriate. Each PedsQL item was reverse-scored and linearly transformed to a 0–100 scale, with higher scores indicating better health-related quality of life. Mean scores were calculated as the sum of item scores divided by the number of items answered. Linear regression analysis was used to determine the relationships between factors, including the number of surgeries and type of surgery, and quality-of-life outcomes. Intraclass correlation coefficient (ICC) analysis was performed to assess the agreement between patients’ quality of life and caregiver-reported quality of life. A *p*-value < 0.05 was considered statistically significant.

## Results

Eighteen patients were diagnosed with craniopharyngioma and were included in this study. The median age at diagnosis was 6.8 years (ranged; 1.7, 13.7). Ten patients (55.6%) were male and 8 patients (44.4%) were female. The median height-SDS and BMI-SDS at diagnosis were –1.6 (–3.2, 0.2) and 0.4 (–1.6, 3.0), respectively. All patients were pre-pubertal at time of diagnosis.

### Clinical presentation and radiologic findings

The most common presenting symptom was increased intracranial pressure, which was documented in 11 patients (61.1%) followed by visual deficits in 6 patients (33.3%) and muscle weakness 1 patient (5.6%). Mixed solid and cystic tumor was the most common type of tumor on imaging, which was documented in 10 patients (55.6%), purely cystic tumor and solid tumors were observed in 7 patients (38.9%) and 1 patient (5.6%), respectively. The median maximal tumor size was 4.3 cm (3.2, 7.5). Hydrocephalus was present in 10 patients (55.6%), and compression of the optic apparatus was identified in 14 patients (77.8%). Initial MRI studies were available for review in 15 patients. Based on the Puget classification [9], 2 patients had no hypothalamic involvement (grade 0), while hypothalamic involvement grade 1 and grade 2 were identified in 4 and 9 patients, respectively (Table 1).

**Table 1:** Clinical characteristics at presentation

Characteristics	*N* = 18
Age at diagnosis (years)	6.8 (1.7, 13.7)
Gender	
Male	10 (55.6)
Female	8 (44.4)
Body weight at diagnosis (kg)	18.3 (7.7, 39.3)
Height at diagnosis (cm)	109.2 (79.5, 144)
Height SDS at diagnosis	−1.6 (−3.2, 0.2)
BMI at diagnosis (kg/m^2^)	15.4 (12.2, 23.0)
BMI SDS at diagnosis	0.4 (−1.6, 3.0)
Male Tanner stage at diagnosis (*n* = 10)	
Genital Tanner stage I	10 (100)
Pubic hair Tanner stage I	10 (100)
Testicular volume (ml)	2 (1, 3)
Female Tanner stage at diagnosis (*n* = 8)	
Breast Tanner stage I	8 (100)
Pubic hair Tanner stage I	8 (100)
Presenting symptoms	
Increase intracranial pressure	11 (61.1)
Visual deficit	6 (33.3)
Muscle weakness	1 (5.6)
Radiographic characteristics of tumor	
Mixed	10 (55.6)
Cystic	7 (38.9)
Solid	1 (5.6)
Maximal tumor size (cm)	4.3 (3.2, 7.5)
Puget classification for hypothalamic involvement	
Grade 0	2 (11.1)
Grade 1	4 (22.2)
Grade 2	9 (50)
N/A	3 (16.7)
Hydrocephalus	10 (55.6)
Compression of the optic apparatus	14 (77.8)
Type of surgery	
Gross total resection	11 (61.1)
Subtotal resection	7 (38.9)
Surgical approach	
Craniotomy	15 (83.3)
Transsphenoidal	3 (16.7)

Data was presented as median (Min, Max) and n (%). Puget classification for hypothalamic involvement: Grade 0 = no hypothalamic involvement; Grade 1 = hypothalamic displacement/compression without invasion; Grade 2 = hypothalamic involvement/invasion. N/A indicates cases with unavailable MRI images for review

### Surgical management and postoperative complications

Eleven patients (61.1%) underwent GTR, the remaining 7 patients (38.9%) underwent STR. Fifteen patients (83.3%) were surgical approach via craniotomy, while 3 patients (16.7%) underwent transsphenoidal approach. Postoperative complications were reported including cerebrospinal fluid leakage in 4 patients (22.2%), presumed infectious meningitis in 3 patients (16.7%), hydrocephalus in 3 patients (16.7%) (Table 2) Ventriculoperitoneal shunt was placed in 10 patients (55.6%). All 18 tumors were classified as adamantinomatous craniopharyngioma on routine histopathological examination using H&E staining; immunohistochemical studies were not performed.

**Table 2:** Pre and Post-operative endocrinologic and surgical complications

Characteristics	*n* = 18
Postoperative complications	
CSF leak	4 (22.2)
Presumed infectious meningitis	3 (16.7)
Hydrocephalus	3 (16.7)
Cranial neuropathies	1 (5.6)
Hypovolemic shock	1 (5.6)
Surgical site bleeding	1 (5.6)
Shunted hydrocephalus	10 (55.6)
Redo surgery	10 (55.6)
Number of tumor removal surgeries	2 (1, 6)
Number of patients requiring ≥ 2 tumor removal surgeries	10 (55.6)
Adjuvant radiation	4 (22.2)
Pathology of tumor: Adamantinomatous	18 (100)
Presurgical pituitary hormone deficit	
GH deficiency	0 (0)
ACTH deficiency	9 (50)
TSH deficiency	7 (38.9)
Vasopressin deficiency	0 (0)
Gonadotropin deficiency	0 (0)
Postsurgical pituitary hormone deficit	
GH deficiency	16 (88.9)
ACTH deficiency	18 (100)
TSH deficiency	18 (100)
Vasopressin deficiency	18 (100)
Gonadotropin deficiency (complete/incomplete)*	14 (93.3) [13/1]

Data was presented as median (Min, Max) and n (%). *Among 15 evaluable patients for pubertal outcome

### Residual/recurrent disease and subsequent management

Overall, 10 of 18 patients (55.6%) developed residual/recurrent disease during follow-up. After the initial surgery, residual/recurrent disease was identified in 7 of 18 patients (38.9%), including 3 of 11 patients (27.3%) who underwent GTR and 4 of 7 patients (57.1%) who underwent STR. The median time to first radiological detection of residual/recurrent disease was 2 months (2, 3) and 3 months (1, 15), respectively.

Overall, 35 tumor resections were performed during the study period, including 25 GTR and 10 STR procedures. Postoperative CT and/or MRI were used to assess residual and recurrent disease, with imaging surveillance tailored according to surgical extent and disease status. Although postoperative imaging was available for clinical follow-up, immediate postoperative MRI suitable for assessment of hypothalamic injury was not routinely obtained. Therefore, postoperative hypothalamic injury could not be reliably graded retrospectively.

**Fig. 1:**
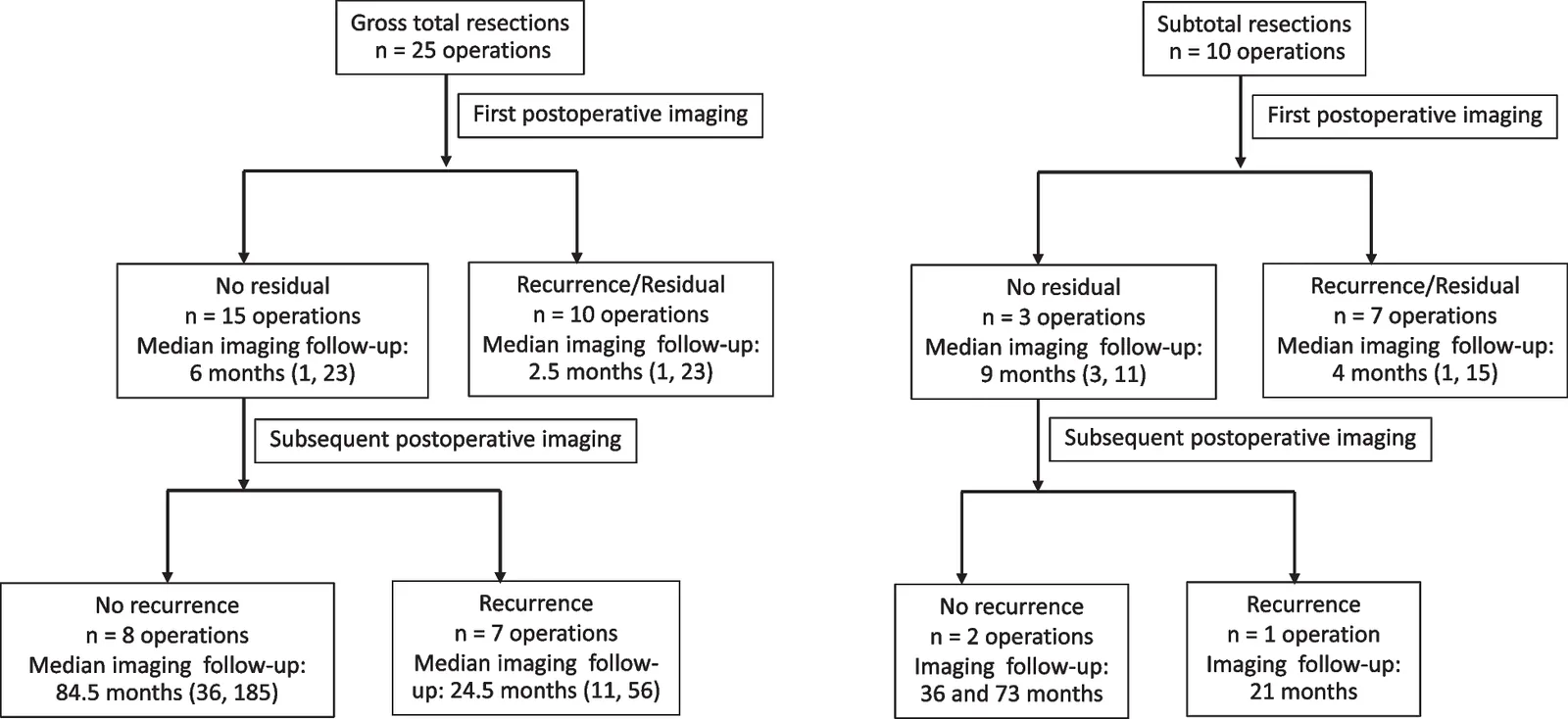
Postoperative radiological outcomes according to extent of resection. Flow diagram showing the proportions of gross total resection (GTR) and subtotal resection (STR) procedures, recurrence/Residual tumor detected on first postoperative imaging, and subsequent recurrence during follow-up. Median imaging follow-up durations are shown for each subgroup

Following GTR, no residual disease was identified on first postoperative imaging after 15 of 25 operations (60.0%). Among these, recurrence subsequently developed after 7 operations, whereas no recurrence was observed after 8 operations. Following STR, no residual disease was identified after 3 of 10 operations (30.0%); recurrence subsequently developed after 1 operation, whereas no recurrence was observed after 2 operations (Fig. 1).

Management strategies for residual/recurrent disease included repeat tumor resection, radiotherapy, cyst aspiration/endoscopic fenestration, Ommaya reservoir placement, and/or intracystic bleomycin therapy, depending on tumor characteristics and location. Ten patients (55.6%) required repeat tumor resection. The median number of tumor removal surgeries was 2 (2, 6). Most tumors with residual/recurrent disease were located in the suprasellar region with hypothalamic involvement. Among the 10 patients with residual/recurrent disease, initial MRI studies were unavailable in 2 patients. Of the remaining 8 patients, the majority demonstrated hypothalamic involvement, with Puget grade 2 identified in 4 patients, compared with grade 1 in 3 patients and grade 0 in 1 patient.

Four patients received radiotherapy (Volumetric Modulated Arc Therapy; VMAT 54 Gy in 27 fractions) for residual/recurrent tumor. One patient with recurrent cystic craniopharyngioma underwent two GTR and one STR, followed by three transsphenoidal surgeries with cyst fenestration and intracystic bleomycin injection for recurrent cystic disease. Ommaya reservoir placement combined with GTR was performed in one patient.

### Presurgical endocrinologic assessments and postsurgical endocrinologic complications and its management

#### Growth hormone (GH)

No patient was documented as having GH deficiency (GHD) before surgery. Postoperatively, GH deficiency was identified in 16 of 18 patients (88.9%). Diagnosis was established based on auxological criteria in all patients, with biochemical confirmation with GH stimulation test which was performed in 13 patients (72.2%), all of whom underwent a clonidine stimulation test. Abnormal peak GH levels (≤ 7 ng/mL) were observed in 92.3% of tested patients. Low insulin-like growth factor 1 (IGF-1) levels were documented in 72.2% of patients. The median peak GH level was 0.06 ng/mL (0.05, 16.6), and the median insulin-like growth factor-1 (IGF-1) level was 37.3 ng/mL (20.7, 181). The median age at GH deficiency diagnosis was 10.5 years (4.4, 15.2), occurring a median interval time of 2.7 years (0.4, 8.8) after surgery. GH therapy was initiated in 9 of 16 patients (56.3%) with GH deficiency. Among the seven untreated patients, one was awaiting radiological confirmation of tumor-free status, three had residual tumor, and three demonstrated preserved linear growth despite evidence of GH deficiency. Notably, all three patients with preserved growth had attained adult height, with a median height SDS of −0.06 (−0.30, 0.60) and a median BMI SDS of 3.0 (2.3, 3.0) at the most recent follow-up.

At the most recent follow-up, three GH-treated and four untreated patients had attained adult height. Median height SDS was −0.82 (−2.58, 2.07) in the GH-treated group and −0.18 (−4.15, 0.60) in the untreated group. Among patients who had not yet completed growth, median height SDS was −3.60 (−3.93, −1.95) in GH-treated patients (*n* = 6) and −2.28 (−4.36, −1.86) in untreated patients (*n* = 3) (Table 2, supplementary table S1).

A representative growth chart illustrating growth deceleration and subsequent response to growth hormone replacement therapy is provided in Supplementary Fig. 1.

### Adrenocorticotropic Hormone (ACTH)

The evaluation for presurgical ACTH deficiency either by morning serum cortisol or 1-mcg ACTH stimulation test was performed in 7 out of 18 patients (38.9%). Two out of 7 patients revealed abnormal test results. The median basal cortisol level of 11.1 µg/dL (0.7, 43.4) and a median peak cortisol level of 26.4 µg/dL (4.9, 35.3). However, all patients received intraoperative hydrocortisone regardless of presurgical test results and continued glucocorticoid replacement therapy postoperatively. Formal postoperative ACTH stimulation testing after temporary glucocorticoid withdrawal was not routinely performed, as most patients had multiple pituitary hormone deficiencies and were considered highly likely to have postoperative ACTH deficiency based on the overall clinical context.

### Thyroid-Stimulating Hormone (TSH)

Prior to surgery, TSH deficiency was screened in 14 patients (77.8%), with abnormal thyroid function tests suggesting central hypothyroidism observed in 7 patients (50%) of evaluated patients. All seven patients with presurgical TSH deficiency continued to have central hypothyroidism postoperatively. After surgery, TSH deficiency was diagnosed in all 18 patients. The mean age at diagnosis of postoperative TSH deficiency was 6.8 years (2.5, 10.8), with a median time to diagnosis of 0.2 months (0.1, 65.0) following surgery. The median TSH level was 1.14 µIU/mL (0.02, 7.0), and the median FT4 level was 0.61 ng/dL (0.3, 1.7). Levothyroxine was administered in all patients.

### Vasopressin (Antidiuretic Hormone)

No patient had documented clinical or biochemical evidence of AVP deficiency before surgery. Presurgical vasopressin deficiency was evaluated in 10 patients (55.6%), and revealed normal laboratory results in all patients tested. Postoperatively, arginine vasopressin deficiency (AVP deficiency) occurred universally, affecting all 18 patients (100%). The median serum sodium of 147.5 mmol/L (137, 159), serum osmolality of 313 mOsmH_2_O/kg (285, 343), and urine osmolality of 102 mOsmH_2_O/kg (57, 388). The median age at diagnosis was 6.5 years (1.7, 12.6), at a median time to diagnosis of 1.0 day (1.0, 2.0) postoperatively. All patients required long-term desmopressin replacement therapy.

### Gonadotropins

No patient was documented as having gonadotropin deficiency before surgery. At the most recent follow-up, 15 patients (83.3%) had reached pubertal age and were evaluated for gonadotropin deficiency/central hypogonadism through assessment of pubertal development and progression, with biochemical confirmation using gonadotropin and sex steroid measurements. The remaining three patients were not evaluated because they had not yet reached the expected age of pubertal onset. Among the 15 evaluated patients, gonadotropin deficiency was documented in 14 patients (93.3%), with absent pubertal development in 13 patients and incomplete pubertal development in 1 patient. The median luteinizing hormone (LH) and follicle-stimulating hormone (FSH) levels were both 0.3 mIU/mL (0.03, 20.4 and 0.3, 8.4, respectively). Median estradiol level was 5.0 pg/mL (5.0, 60.4). All evaluated male patients had testosterone levels less than 2.5 ng/dL, indicating hypogonadotropic hypogonadism. The median age at diagnosis of postoperative gonadotropin deficiency was 13.9 years (11.0, 18.5), occurring a median duration of 6.7 years (1.8, 14.8) after surgery.

### Metabolic complications

Metabolic screening was performed in 16 of 18 patients (88.9%); two patients were not evaluated because they were prepubertal at the time of assessment. The median age at first metabolic screening was 11.1 years (6.3, 19.1), corresponding to a median interval of 4.3 years (0.8, 15.5) after surgery.

During follow-up, 12 patients (66.7%) developed obesity at some point after surgery. The median age at obesity diagnosis was 7.2 years (2.5, 17.8), occurring a median of 1.0 year (0.8, 8.1) after surgery. At diagnosis, most patients were of normal weight (13/18, 72.2%), with 22.2% (4/18) overweight and 5.6% (1/18) obese. At last follow-up, the proportion with normal weight declined to 44.4% (8/18), while overweight and obesity increased to 33.3% (6/18) and 22.2% (4/18), respectively, reflecting a shift toward higher BMI categories over time.

Among screened patients, type 2 diabetes mellitus was diagnosed in one patient (6.3%) at 11.9 years of age, based on an HbA1c level of 6.8%. The median interval of 5.1 years after surgery. No cases of hypertension were identified during follow-up. Dyslipidemia was detected in 10 of 16 screened patients (62.5%), with a median age at diagnosis of 10.5 years (6.3, 14.1) and a median onset of 3.9 years (0.8, 9.9) after surgery. Among patients with dyslipidemia, the median serum triglyceride level was 188 mg/dL (151, 299), and the median low-density lipoprotein (LDL) cholesterol level was 146.3 mg/dL (133.6, 163). MASLD was diagnosed in four patients (25%), at a median age of 10.5 years (8.7, 17.8) and a median of 4.4 years (1.5, 8.8) postoperatively (Table 3).

**Table 3:** Metabolic complications

Metabolic complications	*n* = 18
Metabolic screening	
No (not evaluated due to prepubertal age)	2 (11.1)
Yes	16 (88.9)
Age at first screening (years)	11.1 (6.3, 19.1)
Duration of first screening after surgery (years)	4.3 (0.8, 15.5)
Overweight at any time during follow-up	10 (55.6)/18
Age at diagnosis (years)	7.6 (1.7, 12.9)
Duration after surgery (months)	12 (1.0, 46.0)
Obesity at any time during follow-up	12 (66.7)/18
Age at diagnosis (years)	7.2 (2.5, 17.8)
Duration after surgery (years)	1.0 (0.8, 8.1)
Type 2 diabetes mellitus (*n* = 16)	1 (6.3)/16
Age at diagnosis (years)	11.9
Duration after surgery (years)	5.1
Hypertension (*n* = 16)	0 (0)
Dyslipidemia (*n* = 16)	10 (62.5)/16
Age at diagnosis (years)	10.5 (6.3, 14.1)
Duration after surgery (years)	3.9 (0.8, 9.9)
MASLD (*n* = 16)	4 (25)/16
Age at diagnosis (years)	10.5 (8.7, 17.8)
Duration after surgery (years)	4.4 (1.5, 8.8)

Data was presented as median (Min, Max) and n (%). Overweight and obesity represent cumulative occurrences during follow-up; individual patients may have been classified as both overweight and obese at different time points

Comparative analyses showed no significant differences were observed between patients who received GH therapy and those who did not with respect to overweight, obesity, dyslipidemia, type 2 diabetes mellitus, or MASLD (Table S2).

### Visual complications

At diagnosis, no patients with available ophthalmologic assessment had documented blindness, although baseline visual assessment data were unavailable in 3 patients (16.7%). During follow-up, blindness in at least one eye was identified in 5 of 18 patients (27.8%) at a median age of 9.9 years (6.2, 13.6), occurring a median of 5.8 years (0.3, 10.9) after surgery.

Decreased visual acuity was documented in 72.2% of patients at diagnosis and persisted in 55.6% at the most recent follow-up. At diagnosis, abnormal visual fields were documented in 9 patients (50.0%), although visual field assessment data were unavailable in 7 patients (38.9%). At the most recent follow-up, abnormal visual fields were identified in 13 patients (72.2%). Strabismus was not observed at diagnosis but developed in 55.6% of patients during follow-up. In most cases, strabismus developed after the onset of optic atrophy and significant visual impairment. Optic neuropathy, diagnosed based on ophthalmologic assessment including visual acuity, relative afferent pupillary defect, and fundoscopic evidence of optic disc pallor or optic atrophy, was documented in 7 patients (38.9%) at diagnosis, although assessment data were unavailable in 5 patients (27.8%). At the most recent follow-up, optic neuropathy was present in 14 patients (77.8%) and remained unavailable for assessment in 1 patient (5.6%) (Table 4).

**Table 4:** Visual complications

Visual complications	At diagnosis	At most recent follow-up
Blindness		
- No	15 (83.3)	13 (72.2)
- Yes	0 (0)	5 (27.8)
- N/A	3 (16.7)	0 (0)
Age at diagnosis (years)		9.9 (6.2, 13.6)
Duration after surgery (years)		5.8 (0.3, 10.9)
Decreased visual acuity		
- No	1 (5.6)	8 (44.4)
- Yes	13 (72.2)	10 (55.6)
- N/A	4 (22.2)	0 (0)
Age at diagnosis (years)		7.0 (2.5, 9.8)
Duration after surgery (years)		1.1 (0.3, 4.2)
Abnormal visual field		
- No	2 (11.1)	5 (27.8)
- Yes	9 (50.0)	13 (72.2)
- N/A	7 (38.9)	0 (0)
Age at diagnosis (years)		9.2 (4.8, 14.8)
Duration after surgery (years)		2.2 (0.1, 7.4)
Strabismus		
- No	15 (83.3)	8 (44.4)
- Yes	0 (0)	10 (55.6)
- N/A	3 (16.7)	0 (0)
Age at diagnosis (years)		6.9 (1.8, 15.6)
Duration after surgery (years)		2.3 (0.1, 9.1)
Optic neuropathy		
- No	6 (33.3)	3 (16.7)
- Yes	7 (38.9)	14 (77.8)
- N/A	5 (27.8)	1 (5.6)
Age at diagnosis (years)		7.3 (2.0, 14.9)
Duration after surgery (years)		1.8 (0.1, 5.9)

Data was presented as median (Min, Max) and n (%). N/A indicates missing ophthalmologic evaluation data

### Longitudinal Changes in Height SDS and BMI SDS

**Fig. 2:**
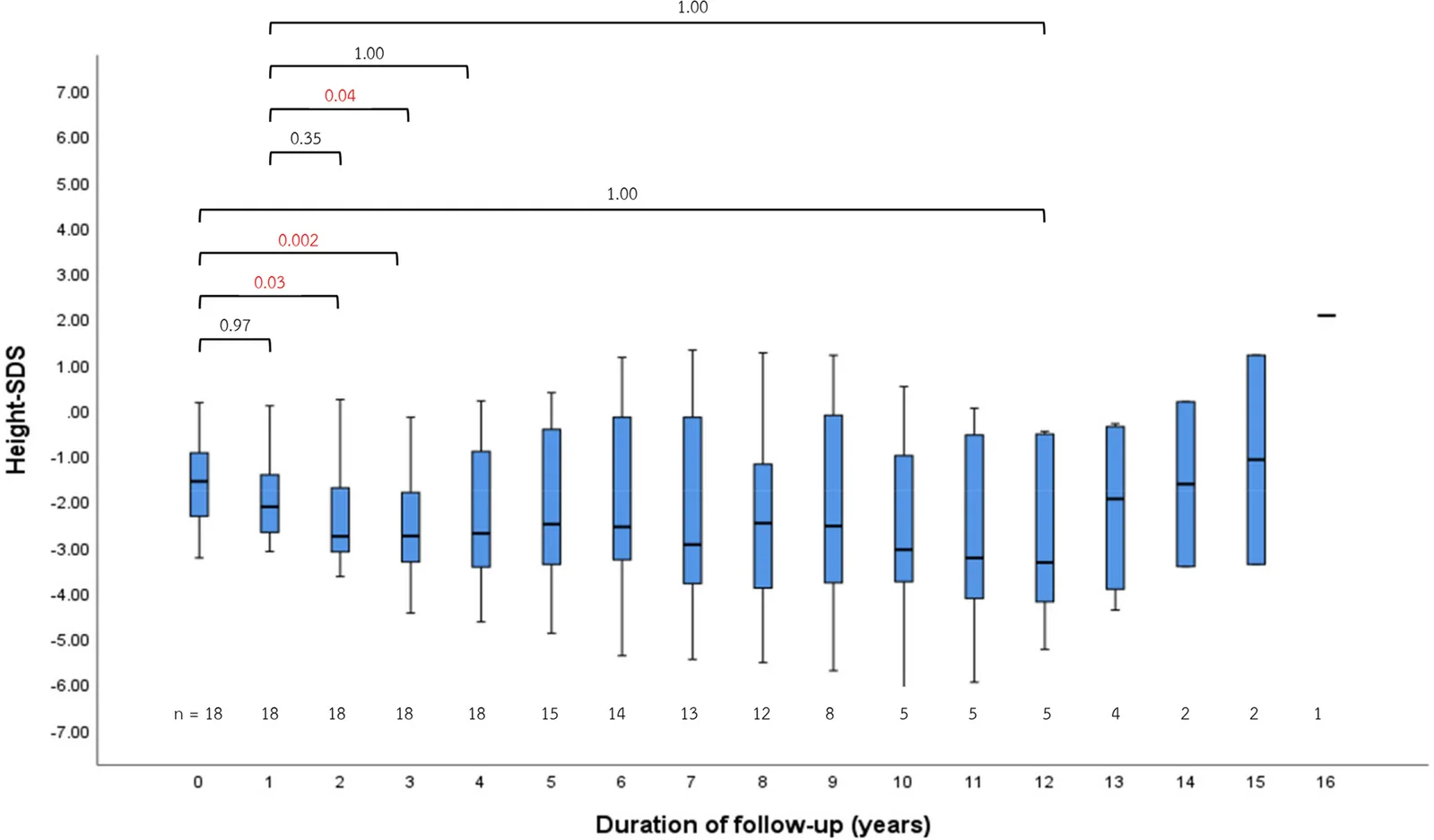
Longitudinal changes in height standard deviation score (Ht-SDS) during follow-up. Box-and-whisker plots illustrate the distribution of Ht-SDS at diagnosis and during follow-up. The p-values represent comparisons with baseline (at diagnosis) values. Numbers below the x-axis indicate the number of patients available at each time point

**Fig. 3:**
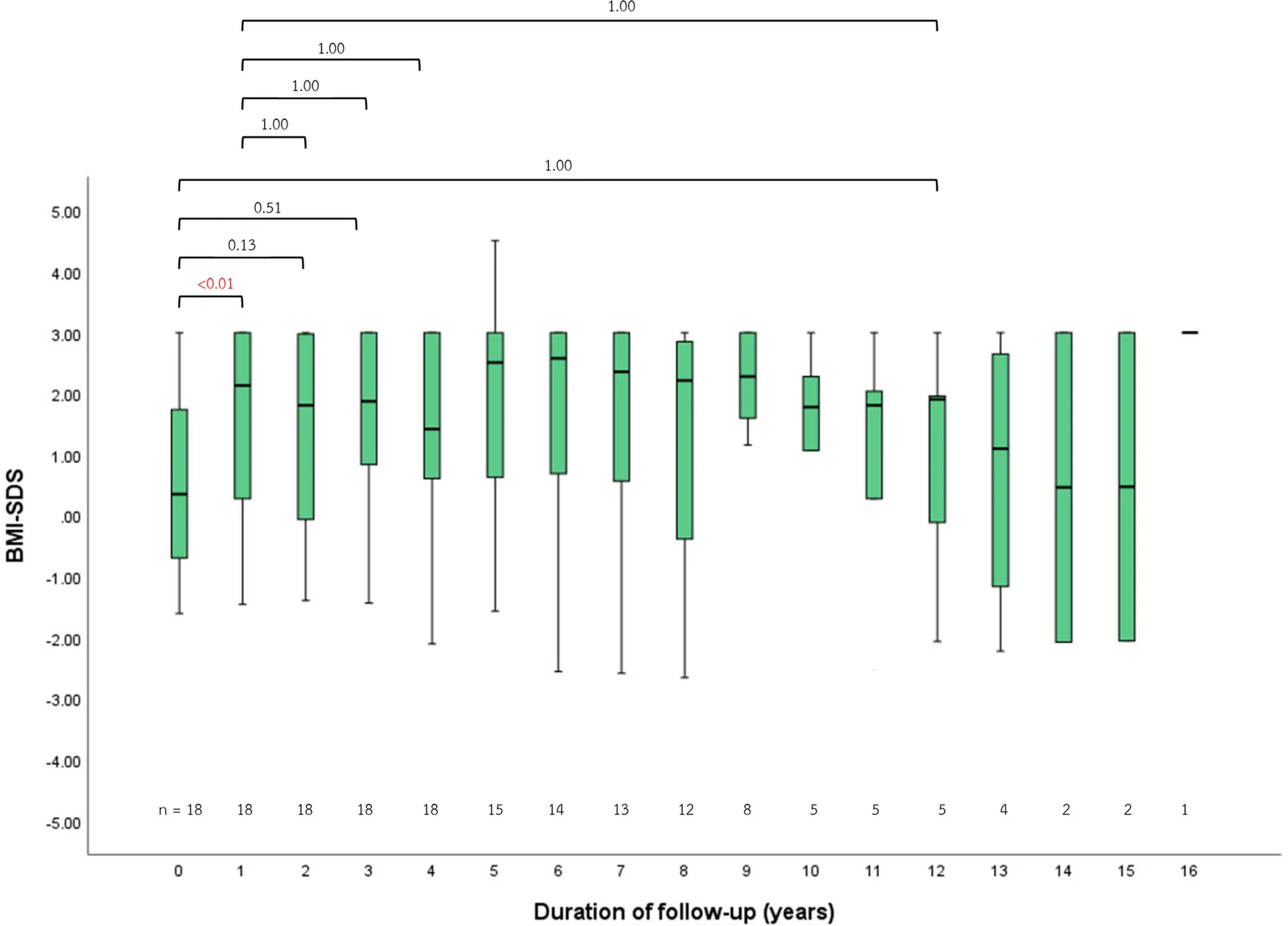
Longitudinal changes in body mass index standard deviation score (BMI SDS) during follow-up. Box-and-whisker plots show BMI SDS at diagnosis and throughout follow-up. BMI SDS increased during the early years after diagnosis and remained elevated during long-term follow-up. The p-values represent comparisons with baseline (at diagnosis) values. Numbers below the x-axis indicate the number of patients available at each time point

Longitudinal analysis on height-SDS BMI-SDS showed a significant decline in height SDS over time following diagnosis and treatment, with values at 2 and 3 years after surgery significantly lower than at diagnosis (all *p* < 0.05). In contrast, BMI SDS increased significantly during follow-up, with a significant rise observed at 1 year compared with diagnosis (*p* < 0.05). These findings indicate impaired linear growth with progressive weight gain (Figs. 2 and 3).

### Quality of life outcomes

Quality of life was assessed using the Pediatric Quality of Life Inventory (PedsQL) across four domains-physical, emotional, social, and school functioning as reported by both patients and their caregivers, stratified by age groups (Table 5).

**Table 5:** PedsQL domains

Age (years)	8–12	13–18	> 18—25	Overall
Patient	*n* = 7	*n* = 6	*n* = 5	*n* = 18
Physical domain	65.2 ± 18.0	66.2 ± 17.5	72.5 ± 17.2	67.5 ± 16.8
Emotional domain	77.1 ± 16.8	75.8 ± 18.3	76.0 ± 11.4	76.4 ± 15.1
Social domain	65.0 ± 15.0	64.2 ± 29.2	75.3 ± 31.9	67.6 ± 24.4
Schooling	67.5 ± 19.2	59.2 ± 24.0	62.3 ± 28.1	63.0 ± 22.5
domain Total	68.4 ± 12.0	66.3 ± 18.6	72.4 ± 17.6	68.8 ± 15.2
Caregiver				
Physical domain	67.4 ± 18.4	53.1 ± 14.5		60.8 ± 17.7
Emotional domain	73.6 ± 15.7	70.0 ± 15.8		71.9 ± 15.2
Social domain	62.9 ± 18.7	56.7 ± 20.4		60.0 ± 18.9
Schooling domain	65.0 ± 20.0	43.3 ± 14.7		54.2 ± 20.2
Total	67.3 ± 14.5	55.4 ± 12.2		61.8 ± 14.3

Data was presented as mean ± SD

### Patient-reported quality of life

The mean total patient-reported PedsQL score for the entire cohort was 68.8 ± 15.2. Among individual domains, physical functioning had a mean score of 67.5 ± 16.8, emotional functioning 76.4 ± 15.1, social functioning 67.6 ± 24.4, and school functioning 63.0 ± 22.5. School functioning consistently demonstrated the lowest scores across age groups. When stratified by age, mean total PedsQL scores were 68.4 ± 12.0 in patients aged 8–12 years, 66.3 ± 18.6 in those aged 13–18 years, and 72.4 ± 17.6 in patients aged 18–25 years.

### Caregiver-reported quality of life

Caregiver-reported total PedsQL scores were generally lower than patient-reported scores, with an overall mean total score of 61.8 ± 14.3. The lowest caregiver-reported scores were observed in the school functioning domain (54.2 ± 20.2). Similar to patient reports, emotional functioning demonstrated relatively higher scores compared with other domains.

### Correlation between patient and caregiver reports

Agreement between patient- and caregiver-reported PedsQL scores was moderate to good overall (ICC for total score = 0.74, *p* = 0.001). Concordance was strongest for emotional (ICC = 0.82) and social (ICC = 0.79) domains and moderate for the physical domain (ICC = 0.64). Age-stratified analysis demonstrated excellent agreement in children aged 8–12 years (total ICC = 0.93), whereas agreement was lower in adolescents aged 13–18 years (total ICC = 0.66), with statistically significant concordance observed primarily in the social domain. No data were available for patients older than 18 years (Table S3).

Overall, caregiver reports tended to align more closely with patient self-reports in younger children than in adolescents, particularly in subjective domains such as physical and school functioning.

### Factors associated with patient-reported quality of life

In univariable linear regression analysis, recent obesity was significantly associated with lower total patient-reported PedsQL scores (B −15.97; 95% CI −30.26 to −1.68; *p* = 0.03). Other factors, including number of surgeries, type of surgery, surgical approach, preoperative hypothalamic involvement, postsurgical pituitary hormone deficiencies, GH replacement therapy, dyslipidemia, type 2 diabetes mellitus, MASLD, and visual complications, were not significantly associated with patient-reported quality of life. Given the small sample size, these analyses should be considered exploratory and no multivariable model was performed to avoid overfitting (Table S4).

### Factors associated with caregiver-reported quality of life

No clinical, endocrine, metabolic, or visual variables were significantly associated with caregiver-reported total PedsQL scores in univariable analysis (Table S5).

## Discussion

This study reviewing data from survivors treated at a tertiary referral center in Thailand confirms the substantial long-term morbidity associated with pediatric craniopharyngioma, with a high prevalence of endocrine, metabolic, visual, and hypothalamic complications, together with an impaired quality of life. Most patients in our cohort demonstrated preoperative hypothalamic involvement, with Puget grade 1 or 2 disease identified in 13 of 15 patients with available MRI studies. Contemporary management increasingly favors hypothalamus-sparing subtotal resection followed by radiotherapy when complete resection carries a high risk of hypothalamic injury [16–18]. However, many patients in our cohort were treated during an era in which GTR was commonly pursued, which may partly explain the high prevalence of endocrine and hypothalamic morbidity observed in our study. The high prevalence of postoperative hypopituitarism observed in our cohort should also be interpreted in the context of tumor characteristics and referral patterns. Most patients presented with substantial local disease burden, including hypothalamic involvement, optic apparatus compression, and hydrocephalus. In addition, as a tertiary referral center, our institution may receive patients with more complex disease, which could have contributed to the severity of endocrine sequelae observed in this cohort. Residual or recurrent disease remained common in our cohort, including after GTR. This underscores the importance of systematic postoperative imaging surveillance. Early postoperative MRI provides an objective assessment of residual disease and serves as an important baseline for subsequent follow-up. Earlier recognition of residual or progressive disease may facilitate timely intervention and help limit further visual, neurological, and endocrine morbidity. Taken together, our findings reinforce the concept that craniopharyngioma is indeed a chronic condition associated with high risk of lifelong sequelae rather than a benign disease with limited long-term consequences [1, 4].

Postoperative pituitary hormone deficiencies were highly prevalent, with TSH and vasopressin deficiencies affecting all patients and GH and gonadotropin deficiencies occurring in the majority. These findings are consistent with previous large series reporting high rates of endocrine dysfunction following treatment, reflecting both tumor-related hypothalamic–pituitary involvement and treatment-associated injury [1–3, 19, 20]. The early onset and persistence of these deficiencies highlight the need for structured, long-term endocrine surveillance and individualized hormone replacement strategies. The endocrine burden was accompanied by substantial metabolic morbidity, which emerged early during follow-up and persisted throughout childhood and adolescence.

Metabolic complications were common and emerged early after surgery, with two-thirds of patients developing obesity and a high prevalence of dyslipidemia and MASLD. This pattern is characteristic of hypothalamic obesity, a well-recognized and challenging consequence of childhood-onset craniopharyngioma [5–7]. The early postoperative onset of obesity observed in this cohort underscores the vulnerability of hypothalamic energy-regulating pathways and the limited effectiveness of conventional lifestyle interventions once hypothalamic damage has occurred. GH therapy in patients with GHD confers benefits beyond linear growth promotion. In addition to increasing height, GH therapy favorably alters body composition by increasing lean body mass while reducing fat mass, changes that are associated with improved cardiometabolic outcomes in individuals with GHD [21, 22]. Importantly, the absence of marked growth failure does not exclude clinically significant GHD in survivors of craniopharyngioma. The phenomenon of “growth without GH” has been described in patients with hypothalamic damage, in whom linear growth may be relatively preserved despite severe GHD [20, 23, 24]. In our cohort, several patients with confirmed GHD did not have substantial height impairment but developed obesity and other metabolic complications during follow-up. These observations suggest that assessment of GHD should extend beyond linear growth alone and include consideration of metabolic health when evaluating the potential benefits of GH replacement therapy. However, in the present study, no significant association was observed between GH therapy and metabolic complications. This finding should be interpreted with caution, as the lack of observed association may be attributable to the limited sample size of the study. Recent evidence suggests that GH therapy may not substantially influence BMI in childhood-onset craniopharyngioma survivors [25]. However, previous studies [26, 27] have reported improvements in lipid profiles, particularly lower LDL cholesterol levels, indicating potential cardiometabolic benefits beyond linear growth promotion.

Craniopharyngioma disrupts the regulation of satiety, reduces energy expenditure, and impairs sympathetic output, resulting in relative parasympathetic predominance. These alterations lead to decreased body temperature, reduced heart rate, and increased daytime sleepiness, ultimately contributing to the development of hypothalamic obesity. This condition is typically refractory to lifestyle modification and may require pharmacologic treatment [2]. Longitudinal analysis on height-SDS and BMI-SDS revealed a discordant pattern of declining height SDS and increasing BMI SDS over time, despite endocrine management. This finding is consistent with prior reports and reflects the combined effects of hypothalamic dysfunction, panhypopituitarism, and treatment-related injury [2, 3]. These results emphasize that optimal endocrine replacement alone may be insufficient to normalize growth trajectories in patients with significant hypothalamic involvement. Despite frequent reports of inadequate response to lifestyle modification, only a small proportion of individuals with hypothalamic obesity had received pharmacological treatment [28, 29]. Social functioning was one of the most affected domains in our cohort. This finding is consistent with previous studies reporting impaired social functioning and poorer quality of life among survivors of childhood-onset craniopharyngioma, particularly in those with obesity and hypothalamic involvement [5, 30]. These findings suggest that effective treatment of hypothalamic obesity may confer broader benefits, including pharmacologic intervention and improvements in social functioning.

Visual morbidity remained prominent, with progressive optic neuropathy and blindness observed during follow-up. Although no patients had documented blindness at diagnosis, more than one-quarter developed unilateral or bilateral blindness later, highlighting the ongoing vulnerability of the optic apparatus even after tumor control [1, 6, 7]. These findings reinforce the importance of long-term ophthalmologic monitoring and early intervention to preserve visual function where possible.

Quality-of-life assessment demonstrated moderate impairment, with school functioning consistently identified as the most affected domain. This is in keeping with prior studies showing that neurocognitive, visual, and endocrine sequelae adversely affect academic performance and social participation in pediatric craniopharyngioma survivors [6, 12, 14, 31]. While obesity was associated with poorer quality of life in univariable analysis, this association did not persist after multivariable adjustment, suggesting that quality of life is influenced by the cumulative burden of multiple chronic complications rather than a single dominant factor. Given the recognized impact of hypothalamic dysfunction on long-term morbidity and quality of life in pediatric-onset craniopharyngioma [3, 9, 32], we reviewed preoperative MRI studies and classified hypothalamic involvement according to the Puget classification. Preoperative hypothalamic involvement was not significantly associated with total PedsQL scores in our exploratory analysis, although this finding should be interpreted cautiously because of the small sample size. Nevertheless, hypothalamic involvement was common among patients with residual or recurrent disease, supporting previous observations that tumors involving the hypothalamic region are more challenging to resect completely and may be associated with less favorable surgical outcomes.

Caregiver-reported quality-of-life scores were generally lower than patient-reported scores, particularly in younger children, with strong concordance observed in the 8 to 12-year age group. This finding aligns with previous literature indicating that caregivers may perceive greater functional impairment, whereas adolescents may underreport difficulties, underscoring the importance of incorporating both patient and caregiver perspectives into routine assessment [12, 14, 31].

The findings of this study highlight the necessity of lifelong, multidisciplinary follow-up for pediatric craniopharyngioma survivors. Early identification and proactive management of endocrine deficiencies, hypothalamic obesity, cardiometabolic risk factors, and visual impairment are essential to mitigate long-term morbidity. Regular assessment of quality of life should be incorporated into clinical care, with particular attention to school functioning and psychosocial well-being. In resource-limited settings, coordinated care pathways and standardized follow-up protocols may help optimize outcomes and reduce preventable complications.

Several limitations should be acknowledged. The retrospective design and small sample size limited statistical power, and some clinical data were unavailable because of incomplete documentation. Missing baseline ophthalmologic data in a proportion of patients may have introduced bias in estimates of visual morbidity and precluded definitive assessment of changes over time. Therefore, visual outcome data should be interpreted with caution. The extent of resection was based on operative reports and available postoperative imaging; however, variation in imaging surveillance and the absence of early postoperative MRI in some patients made distinction between residual and recurrent disease difficult in certain cases. In addition, postoperative hypothalamic injury could not be systematically assessed because standardized postoperative MRI suitable for hypothalamic grading was not routinely available. As a single-center study, the findings may not be fully generalizable to other populations.

## Conclusions

Pediatric craniopharyngioma is associated with substantial long-term endocrine, metabolic, visual, and quality-of-life impairments. These findings underscore the need for lifelong, multidisciplinary care focused not only on tumor control but also on optimizing functional outcomes and quality of life in this vulnerable population.

## Supplementary Information

Supplementary Material 1

Supplementary Material 2

## Data Availability

The datasets used and/or analysed during the current study are available from the corresponding author on reasonable request.
