# Supplementary material for: Long-term endocrine complications and quality of life in pediatric-onset craniopharyngioma: a single-center cohort study
Source: BMC Pediatr. 2026 Jul 15;26:976. doi: 10.1186/s12887-026-07309-0 (PMC13656672; doi:10.1186/s12887-026-07309-0)
Supplement: Supplementary file 1 [file 12887_2026_7309_MOESM1_ESM.pdf]

Girls aged 2-19 years: Height and Weight

THAI SOCIETY for PEDIATRIC ENDOCRINOLOGY

Name____________ DOB____________ HN____________

Father's height 173 cm, Mother's height 153 cm, Mid-parental height 156.5 cm

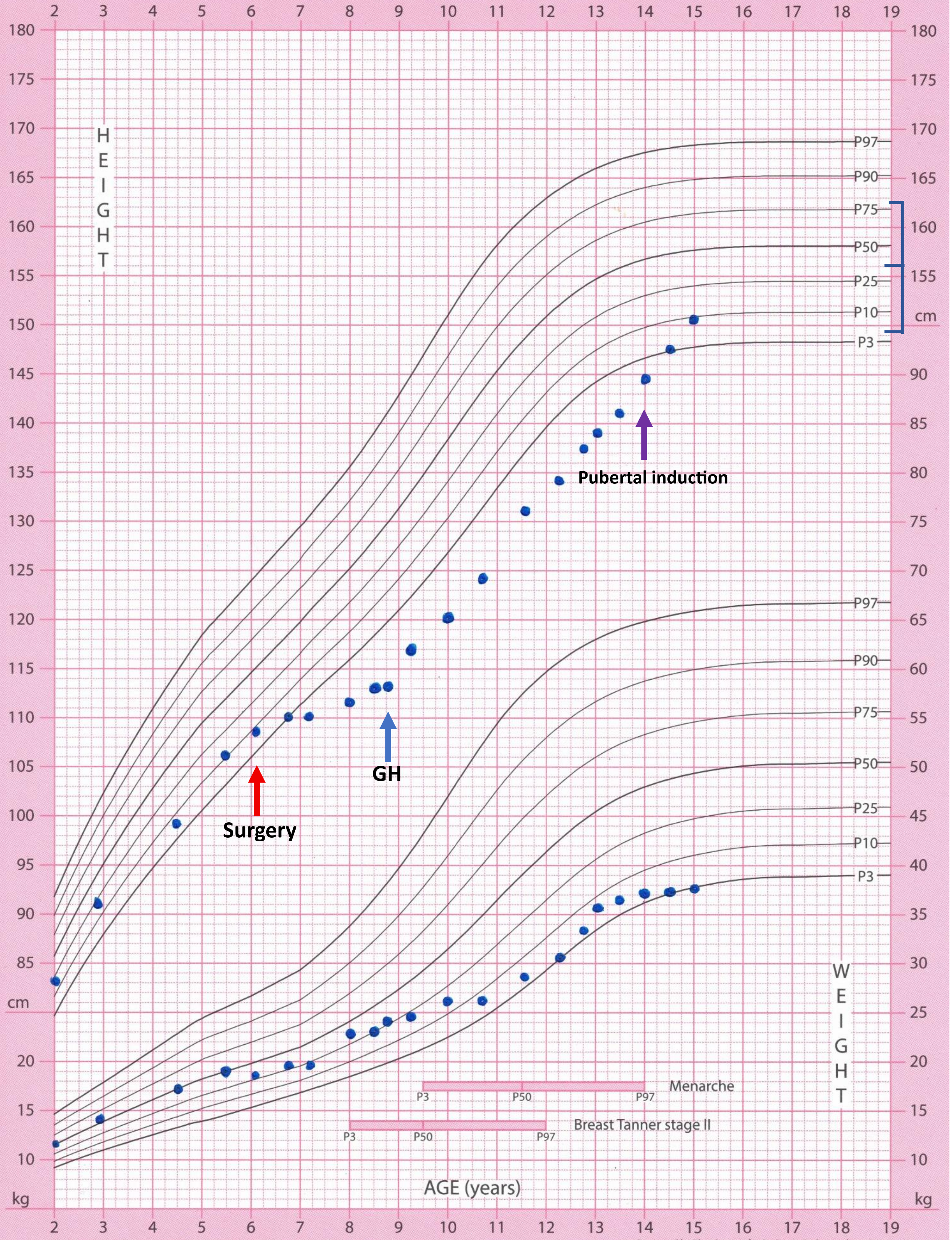

References: WHO Growth Standard for children aged 2-5 years, 2006
National Growth References for children aged 5-19 years, 2020, Bureau of Nutrition, Department of Health: Ministry of Public Health

Designed by Thai Society for Pediatric Endocrinology, 2022
