# Supplementary material for: Long-term endocrine complications and quality of life in pediatric-onset craniopharyngioma: a single-center cohort study
Source: BMC Pediatr. 2026 Jul 15;26:976. doi: 10.1186/s12887-026-07309-0 (PMC13656672; doi:10.1186/s12887-026-07309-0)
Supplement: Supplementary file 2 [file 12887_2026_7309_MOESM2_ESM.docx]

**Supplementary Tables**

**Table S1. Pre and post-surgical pituitary hormone deficits and their management**

| **Characteristics** | **n=18** |
| --- | --- |
| **Presurgical pituitary hormone deficit** | **n=18** |
| GH deficiency  - Evaluation  No  Yes | 18 (100)  0 (0) |
| ACTH deficiency  - Evaluation  No  Yes  - Laboratory result  Normal  Abnormal | 11 (61.1)  7 (38.9)  5 (71.4)  2 (28.6) |
| TSH deficiency  - Evaluation  No  Yes  - Laboratory result  Normal  Abnormal | 4 (22.2)  14 (77.8)  7 (50)  7 (50) |
| Vasopressin deficiency  - Evaluation  No  Yes  - Laboratory result  Normal  Abnormal | 8 (44.4)  10 (55.6)  10 (100.0)  0 (0) |
| GnRH deficiency  - Evaluation  No  Yes | 18 (100)  0 (0) |
| **Postsurgical pituitary hormone deficit** | **n=18** |
| GH deficiency  - Diagnosis by clinical (auxological data)  - Diagnosis by biochemical data  - Evaluation of GH stimulation test  No  Yes  - Clonidine test  - Laboratory result: peak GH < 7 ng/mL)  - IGF-1 level  Low  Normal  High  N/A  - GH replacement therapy  - Age at diagnosis (years)  - Duration after surgery (years) | 18 (100)  12 (66.7)  5 (27.8)  13 (72.2)  13 (100)  13 (100)  13 (72.2)  2 (11.1)  0  3 (16.7)  9 (50)   10.5 (4.4, 15.2)   2.7 (0.4, 8.8) |
| ACTH deficiency  - Evaluation of 1 mcg ACTH stimulating test after surgery  No  Yes  - Received glucocorticoid replacement  - Age at diagnosis (years) | 18 (100)  0 (0)  18 (100)  6.8 (1.7, 12.6) |
| TSH deficiency  - Evaluation  No  Yes  - Laboratory result  Normal  Abnormal  - Age at diagnosis (years)  - Duration after surgery (months) | 0 (0)  18 (100)  0 (0)  18 (100)   6.8 (2.5, 10.8)   0.2 (0.1, 65) |
| Vasopressin deficiency  - Evaluation  No  Yes  - Laboratory result  Normal  Abnormal  - Age at diagnosis (years)  - Duration after surgery (days) | 0 (0)  18 (100)  0 (0)  18 (100)  6.5 (1.7, 12.6)  1.0 (1.0, 2.0) |
| GnRH deficiency  - Evaluation  Yes  No (not evaluated due to prepubertal age)  - Laboratory result  Normal pubertal development  Absent of pubertal development  Incomplete pubertal development  - Male  LH (IU/L)  FSH (IU/L)  Testosterone (ng/dL)  - Female  LH (IU/L)  FSH (IU/L)  Estradiol (pg/mL)  - Age at diagnosis (years)  - Duration after surgery (years) | 15 (83.3)  3 (16.7)  1 (6.7)  13 (86.7)  1 (6.7)  0.3 (0.03, 1.05)  0.3 (0.30, 1.66)  2.50 (2.50, 2.50)  0.52 (0.03, 20.40)  1.42 (0.30, 8.36)  5.00 (5.00, 60.40)  13.9 (11.0, 18.5)  6.7 (1.8, 14.8) |
| Presurgical hormone replacement therapy  Growth hormone  Glucocorticoid  Levothyroxine  Desmopressin  Sex hormone | 0 (0)  2 (11.1)  7 (38.9)  0 (0)  0 (0) |
| Postsurgical hormone replacement therapy  Growth hormone  Glucocorticoid  Levothyroxine  Desmopressin  Sex hormone | 9 (50)  18 (100)  18 (100)  18 (100)  11 (61.1) |

Data was presented as median (Min, Max) and n (%). Abbreviation: GH, growth hormone; ACTH, adrenocorticotropic hormone; TSH, thyroid-stimulating hormone; GnRH, gonadotropin-releasing hormone; IGF-1, insulin-like growth factor 1; N/A, not available.

**Table S2: Association Between Growth Hormone Replacement Therapy and Metabolic Complications**

| **Metabolic complications** | **Received GH replacement therapy** | **Did not receive GH replacement therapy** | **OR (95%CI)** | **p-value** |
| --- | --- | --- | --- | --- |
| Overweight | 5 (55.6) | 5 (55.6) | 1 (0.16, 6.42) | 1.00 |
| Obesity | 4 (44.4) | 8 (88.8) | 0.10 (0.01, 1.17) | 0.13 |
| Type 2 diabetes mellitus | 1 (11.1) | 0 (0) | N/A | 1.00 |
| Dyslipidemia | 4 (44.4) | 6 (66.7) | 0.40 (0.06, 2.70) | 0.64 |
| MASLD | 3 (33.3) | 4 (44.4) | 0.63 (0.09, 4.22) | 1.00 |

Data are presented as n (%). Odds ratios were calculated using univariable logistic regression.

**Table S3: Correlations between Patient’s and Caregiver’s PedsQL**

| **QoL reporter** | **Intraclass correlation coefficient** | **95%CI p-value** |
| --- | --- | --- |
| Overall  - Physical domain  - Emotional domain  - Social domain  - Schooling domain  - Total | 0.64  0.82  0.79  0.74  0.74 | 0.16, 0.87 0.01  0.94, 0.94 < 0.001  0.45, 0.93 < 0.001  0.32, 0.92 0.002  0.35, 0.91 0.001 |
| Patient age 8-12 yr  - Physical domain  - Emotional domain  - Social domain  - Schooling domain  - Total | 0.95  0.94  0.65  0.99  0.93 | 0.78, 0.99 < 0.001  0.70, 0.99 < 0.001  -0.10, 0.93 0.04  0.93, 1.00 < 0.01  0.65, 0.10 < 0.01 |
| Patient age 13-18 yr  - Physical domain  - Emotional domain  - Social domain  - Schooling domain  - Total | 0.36  0.69  0.88  0.53  0.66 | -0.54, 0.88 0.21  -0.14, 0.95 0.05  0.36, 0.98 0.01  -0.38, 0.92 0.11  -0.19, 0.94 0.05 |
| Patient age 18-25 yr | N/A | N/A |

Agreement between patient- and caregiver-reported PedsQL scores was assessed using the intraclass correlation coefficient (ICC).

**Table S4: Factors that associated with total PedsQL of patients**

| **Factors** | **Univariable analysis** |
| --- | --- |
|  | **B (95% CI) p-value** |
| Number of surgery | -4.51 (-9.64, 0.61) 0.08 |
| Type of surgery  Subtotal resection | 1.17 (-14.88, 17.22) 0.88 |
| Surgical approach  Transsphenoid | 6.56 (-14.17, 27.28) 0.51 |
| Postsurgical GH deficiency | -9.77 (-34.14, 14.60) 0.41 |
| Postsurgical GnRH deficiency | -5.08 (-23.72, 13.56) 0.57 |
| Received GH replacement therapy | 0.68 (-14.98, 16.33) 0.93 |
| Recent obesity | -15.97 (-30.26, -1.68) 0.03 |
| Recent T2DM | 11.16 (-22.51, 44.83) 0.49 |
| Recent dyslipidemia | -13.27 (-27.37, 0.83) 0.06 |
| Recent MASLD | -5.93 (-21.68, 9.82) 0.44 |
| Recent blindness | -7.91 (-24.88, 9.07) 0.34 |
| Recent decrease visual acuity | -1.93 (-17.65, 13.80) 0.80 |
| Recent abnormal visual field | -8.59 (-25.46, 8.29) 0.30 |
| Recent strabismus | -7.86 (-23.06, 7.34) 0.29 |
| Recent optic neuropathy | -13.70 (-32.30, 4.90) 0.14 |
| Preoperative hypothalamic involvement | -8.41 (-31.87, 15.05) 0.45 |

Results are from exploratory univariable linear regression analyses. Estimates should be interpreted with caution because of the small sample size and missing data for some variables. Preoperative hypothalamic involvement was classified according to the Puget classification.

**Table S5: Factors that associated with total PedsQL of caregivers**

| **Factors** | **Univariable analysis** |
| --- | --- |
|  | **B (95% CI) p-value** |
| Number of surgery | -3.46 (-9.10, 2.17) 0.20 |
| Type of surgery  Subtotal resection | 1.35 (-18.41, 21.11) 0.88 |
| Surgical approach  Transsphenoid | 3.45 (-18.10, 24.99) 0.73 |
| Postsurgical GH deficiency | N/A |
| Postsurgical GnRH deficiency | -3.74 (-25.26, 17.79) 0.71 |
| Received GH replacement therapy | 9.00 (-8.31, 26.31) 0.28 |
| Recent obesity | -12.33 (-30.34, 5.67) 0.16 |
| Recent T2DM | 21.32 (-9.88, 52.52) 0.16 |
| Recent dyslipidemia | -4.59 (-22.65, 13.47) 0.59 |
| Recent MASLD | 0.01 (-19.77, 19.79) 1.00 |
| Recent blindness | -5.48 (-24.92, 13.96) 0.55 |
| Recent decrease visual acuity | 1.25 (-17.50, 20.00) 0.89 |
| Recent abnormal visual field | -11.92 (-32.10, 8.25) 0.22 |
| Recent strabismus | 2.64 (-16.04, 21.32) 0.76 |
| Recent optic neuropathy | -11.92 (-32.09, 8.25) 0.22 |
| Preoperative hypothalamic involvement | 13.66 (-9.59, 36.92) 0.22 |

Results are from exploratory univariable linear regression analyses. Estimates should be interpreted with caution because of the small sample size and missing data for some variables. Preoperative hypothalamic involvement was classified according to the Puget classification.
